# Fluorescent pulse-chase labeling to monitor long-term mitochondrial degradation in primary hippocampal neurons

**DOI:** 10.1016/j.xpro.2022.101822

**Published:** 2022-11-29

**Authors:** Jordan R. Schneider, Chantell S. Evans

**Affiliations:** 1 Department of Cell Biology, Duke University Medical Center, Durham, NC 27710, USA

**Keywords:** Cell Biology, Cell culture, Cell-based Assays, Metabolism, Microscopy, Molecular Biology, Neuroscience, Molecular/Chemical Probes

## Abstract

The accumulation of dysfunctional mitochondria is a hallmark of neurodegenerative diseases, yet the dynamics of mitochondrial turnover in neurons are unclear. Here, we describe a protocol to monitor the degradation of spectrally distinct, “aged” mitochondrial populations. We describe the preparation and transfection of primary rat hippocampal neuron cultures. We detail a mitochondrial-damaging assay, a SNAP pulse-chase labeling paradigm, and live imaging to visualize the mitochondrial network. Finally, we provide steps to quantify mitochondrial turnover via lysosomal fusion.

For complete details on the use and execution of this protocol, please refer to Evans and Holzbaur (2020a).

## Highlights

• Primary hippocampal cultures to monitor long-term neuronal mitochondrial degradation
• Inducing mitochondrial damage through global antioxidant deprivation
• A fluorescent pulse-chase protocol to visualize “aged” mitochondrial populations
• Live imaging and quantitative analysis of mitochondrial turnover via lysosomal fusion

## Abstract (editor highlights)

Publisher’s note: Undertaking any experimental protocol requires adherence to local institutional guidelines for laboratory safety and ethics.

## Graphical abstract

**Figure:**
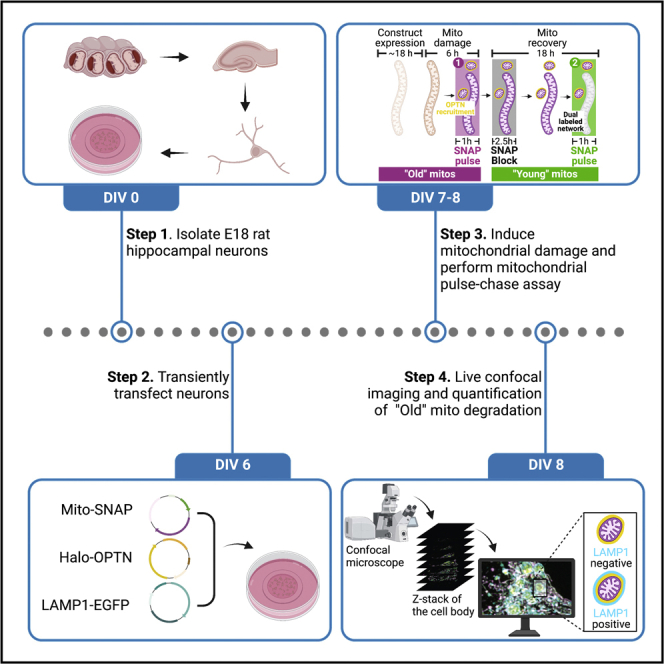

## Before you begin

The protocol below outlines steps necessary for pulse-chase labeling of “aged” neuronal mitochondria to investigate long-term degradation following organelle damage. Here, we describe reagents, equipment, and experimental steps for 1) preparing and maintaining primary hippocampal cultures, 2) transient transfection of neurons, 3) mitochondrial damaging and pulse-chase labeling assays, and 4) confocal imaging and analysis of mitochondrial turnover in neurons. A timed-pregnant Sprague-Dawley wild-type rat should be purchased ahead to ensure the dissection is performed on embryonic day 18 (E18). Primary hippocampal cultures require media exchanges or “feedings” every 3–4 days to maintain culture health. Neuronal cultures are maintained for six days before transient transfection and pulse-chase labeling.

Before starting the experiment, several buffers and media should be prepared. See the materials and equipment and key resources table for detailed information.

### Institutional permissions

The animals used in this study are approved by the Duke Institutional Animal Care and Use Committee (#A230-21-11). Others who wish to replicate this protocol should seek approval from their respective institutions.

### Coating glass imaging dishes

**Timing: 18 h**

Poly-L-Lysine (PLL) promotes the adhesion of primary neurons to the glass surface of imaging dishes. It is added overnight (∼14–16 h) to coat the glass surface.

1. Add 1 mL of 0.5 mg/mL PLL to the glass surface of the imaging dishes.
2. Incubate ∼14–16 h at 37°C in a 5% CO_2_ incubator.
3. Aspirate PLL and wash the coated surface twice with 2 mL of sterile ddH_2_O.
4. Wash coated surface twice with 2 mL of Neurobasal media.
5. Add 2 mL of Attachment Media (AM).
6. Place dishes in the 5% CO_2_ incubator at 37°C to allow AM to warm and equilibrate. Store dishes in the incubator until ready to plate neurons.

### Dissection preparation

**Timing: 30 min**

Prior to beginning the dissection, media should be prepared and incubated to the appropriate temperature. When performing the dissection and preparing primary cultures, it is essential to use sterile technique to avoid contamination.

7. Prepare AM and Maintenance Media (MM) for primary hippocampal cultures.
8. Prewarm 5 mL of HBSS and 35 mL of AM in a 37°C water bath.
9. Place a 15 mL conical containing 10 mL of HBSS on ice.
10. Sterilize scissors, forceps, and dissection area with 70% ethanol.

## Key resources table

**Table:** 

REAGENT or RESOURCE	SOURCE	IDENTIFIER
**Chemicals, peptides, and recombinant proteins**		

AraC (Cytosine β-D-arabinofuranoside hydrochloride)	Sigma-Aldrich	C6645
Boric Acid	Gibco	A73-500
B27 Supplement (50×)	Gibco	17504-044
B27 Supplement (50×), minus antioxidants	Gibco	10889-038
Glucose (45%)	Sigma-Aldrich	G8768-100
GlutaMAX Supplement (100×)	Gibco	35050-061
HBSS (10×)	Gibco	14185-052
HEPES (1M)	Gibco	15630-080
Horse Serum, New Zealand Origin	Gibco	16050122
Minimum Essential Media (MEM) (1×)	Corning	10-010-CV
Neurobasal Media	Gibco	21103-049
Penicillin/Streptomycin (100×)	Gibco	15140-122
Poly-L-Lysine Hydrobromide	Sigma-Aldrich	P1274
Pyruvic Acid	Corning	25-000-CI
Sodium Tetraborate Decahydrate	Fisher Scientific	S248-500
Lipofectamine 2000 Transfection Reagent	Invitrogen	11668019
Trypsin (2.5%), no phenol red	Gibco	15090046
Trypan Blue Stain, 0.4%	Invitrogen	T10282
DMSO, Anhydrous	Molecular Probes	D12345
JaneliaFluor SNAP JF549cp	Janelia	N/A
SNAP-Cell 430	New England Biolabs	S9109S
SNAP-Cell Block	New England Biolabs	S9106S
JaneliaFluor Halo JF646x	Janelia	N/A
Zymo Research ZymoPURE II Plasmid Maxiprep Kit	Genesee	11-555B

**Experimental models: Organisms/strains**		

Rat: CD (Sprague Dawley) IGS, wild-type, E18 Timed-Pregnant, female	Charles Rivers	RGD: RRID: RGD_737891; Crl:CD(SD); Strain Code: 001

**Recombinant DNA**		

Mito-SNAP	Moore and Holzbaur (2016)	N/A
Halo-OPTN	Moore and Holzbaur (2016)	N/A
LAMP1-EGFP	Evans and Holzbaur (2020a)	N/A

**Software and algorithms**		

Fiji	(Schindelin, 2012)	https://imagej.net/software/fiji/
TurboReg	(Thevenaz, 1998)	http://bigwww.epfl.ch/thevenaz/turboreg/
Leica Application Suite X (LAS X)	Leica	https://www.leica-microsystems.com/products/microscope-software/p/leica-las-x-ls/
Prism9	GraphPad Software	https://www.graphpad.com
Adobe Illustrator	Adobe Inc.	https://www.adobe.com/products/illustrator

**Other**		

S9i Stereo Microscope	Leica	N/A
DMiL Inverted Tissue Culture Microscope	Leica	N/A
STELLARIS 8 Confocal Microscope	Leica	N/A
LIGHTNING Deconvolution Software	Leica	N/A
Vannas Tubigen Spring Scissors	Fine Science Tools	15003-08
Dumont #5SF Forceps	Fine Science Tools	11252-00
Surgical Scissors	Fine Science Tools	14001-16
Fine Scissors - Sharp	Fine Science Tools	1460-09
35 mm Glass Bottom MatTek Dishes	MatTek	P35G-1.2-20-C

## Materials and equipment

**Table:** Borate Buffer

Reagent	Final concentration	Amount
Boric Acid	50 mM	1.2366 g
Sodium Tetraborate Decahydrate	12.5 mM	1.9 g
ddH_2_O	N/A	400 mL
**Total**	**N/A**	**400 mL**

• Preparation procedure: Dissolve boric acid and sodium tetraborate in 400 mL of ddH_2_O. Filter buffer using a 0.22 μm filter unit to sterilize and if necessary, adjust pH to 8.7–9.0.
• Stock solution can be stored for ∼4 months at 4°C.

**CRITICAL:** Boric Acid and Sodium Tetraborate are Particularly Hazardous chemicals. Store in a cool, dry, well-ventilated area away from incompatible materials. Wear proper PPE when working with chemicals and know institutional approved procedures for cleaning spills.

**Table:** Poly-L-Lysine

Reagent	Final concentration	Amount
Poly-L-Lysine (PLL)	2 mg/mL	100 mg
Borate Buffer	1×	50 mL
**Total**	**N/A**	**50 mL**

• Preparation procedure: Dissolve 100 mg of PLL in 50 mL of borate buffer. Make 1 mL aliquots and store at −80°C for up to ∼3 months.
• For each experiment, thaw aliquot(s) and dilute to 0.5 mg/mL with sterile ddH_2_O.

**Table:** 1× HBSS

Reagent	Final concentration	Amount
10× HBSS	1×	50 mL
1M HEPES	10 mM	5 mL
ddH_2_O	N/A	445 mL
**Total**	**N/A**	**500 mL**

• Preparation procedure: Filter medium using a 0.22 μm filter unit to sterilize.
• If making 1M HEPES from powder, adjust pH to 7.4.
• Solution is stored at 4°C for ∼2 months.

**Table:** Attachment Media (AM)

Reagent	Final concentration	Amount
Minimum Essential Medium (MEM)	1×	43.4 mL
Heat Inactivated Horse Serum	10%	5 mL
Pyruvic Acid	1 mM	0.5 mL
45% Glucose	33 mM	0.66 mL
**Total**		**50 mL**

• Preparation procedure: Filter the medium using a 0.22 μm filter unit to sterilize.
• Store at 4°C for 2–3 weeks.
• Horse serum is inactivated by heating to 56°C for 30 min.

**Table:** Maintenance Media (MM)

Reagent	Final concentration	Amount
Neurobasal Media	1×	47.34 mL
GlutaMAX Supplement	2 mM	0.5 mL
Penicillin/Streptomycin	1×	0.5 mL
45% Glucose	33 mM	0.66 mL
B-27 Supplement	2%	1 mL
**Total**		**50 mL**

• Preparation procedure: Filter the medium using a 0.22 μm filter unit to sterilize and then add B-27 supplement.
• MM can be stored at 4°C for 3–4 weeks.

**Table:** Antioxidant free Maintenance Media (AOF MM)

Reagent	Final concentration	Amount
Neurobasal Media	1×	47.34 mL
GlutaMAX Supplement	2 mM	0.5 mL
Penicillin/Streptomycin	1×	0.5 mL
45% Glucose	33 mM	0.66 mL
B-27 Supplement, minus antioxidants	2%	1 mL
**Total**		**50 mL**

• Preparation procedure: Filter the medium using a 0.22 μm filter unit to sterilize and then add B-27, minus antioxidants supplement.
• The B-27 supplement, minus antioxidants does not contain vitamin E, vitamin E acetate, superoxide dismutase, catalase, and glutathione.
• AOF MM can be stored at 4°C for 3–4 weeks.

**Table:** AraC Stock Solution

Reagent	Final concentration	Amount
AraC	25 mM	100 mg
ddH_2_O	N/A	14.3 mL
**Total**		**14.3 mL**

• Preparation procedure: Dissolve AraC in ddH_2_O. Aliquot into 0.5 mL and store at −20°C for up to 4 months.

**Table:** Stock solution: SNAP (JF549cp, 430, and Block) and Halo (JF646x) Ligands

Reagent	Final concentration	Amount
SNAP or Halo Ligands	1 mM	50 or 100 nmol
DMSO	N/A	50 or 100 μL
**Total**		**50 or 100 μL**

• Preparation procedure: Dissolve SNAP or Halo substrate in DMSO and vortex for 10 min. Prepare 2 μL aliquots in amber tubes and store in the dark at −20°C for 6 months.
• Ligands come as 50 or 100 nmol and should be diluted to a final concentration of 1 mM with DMSO.

**Table:** Working solution: SNAP (JF549cp) and Halo (JF646x) Dilutions

Reagent	Final concentration	Preparation procedure
SNAP JF549cp (1 mM stock)	20 μM	Make a working solution by mixing 2 μL of stock solution with 98 μL of MM. Thoroughly mix.
Halo JF646x (1 mM stock)	20 μM	Make a working solution by mixing 2 μL of stock solution with 98 μL of MM. Thoroughly mix.

• Working solution can be stored in the dark at 4°C for 2 months. Stock should be prepared in amber tubes.
• Stock solution = 1 mM; Working solution = 20 μM.

**Table:** SNAP and Halo Labeling

Reagent	Final concentration	Preparation procedure
SNAP JF549cp (20 μM)	100 nM	To each imaging dish, add 10 μL of working solution in 2 mL of media.
SNAP-Cell 430 (1 mM)	2 μM	To each imaging dish, add 1 μL of stock solution in 500 μL of media.
SNAP-Cell Block (1 mM)	1 μM	To each imaging dish, add 1 μL of stock in 1 mL of media.
Halo JF646x (20 μM)	100 nM	To each imaging dish, add 10 μL of working solution in 2 mL of media.

## Step-by-step method details

### Tissue harvesting

**Timing: 15 min**

Harvesting embryos from an E18 timed-pregnant rat should be performed quickly to maintain high-quality tissue. It is important to use fresh forceps and scissors between steps to prevent contamination.

**Figure 1:**
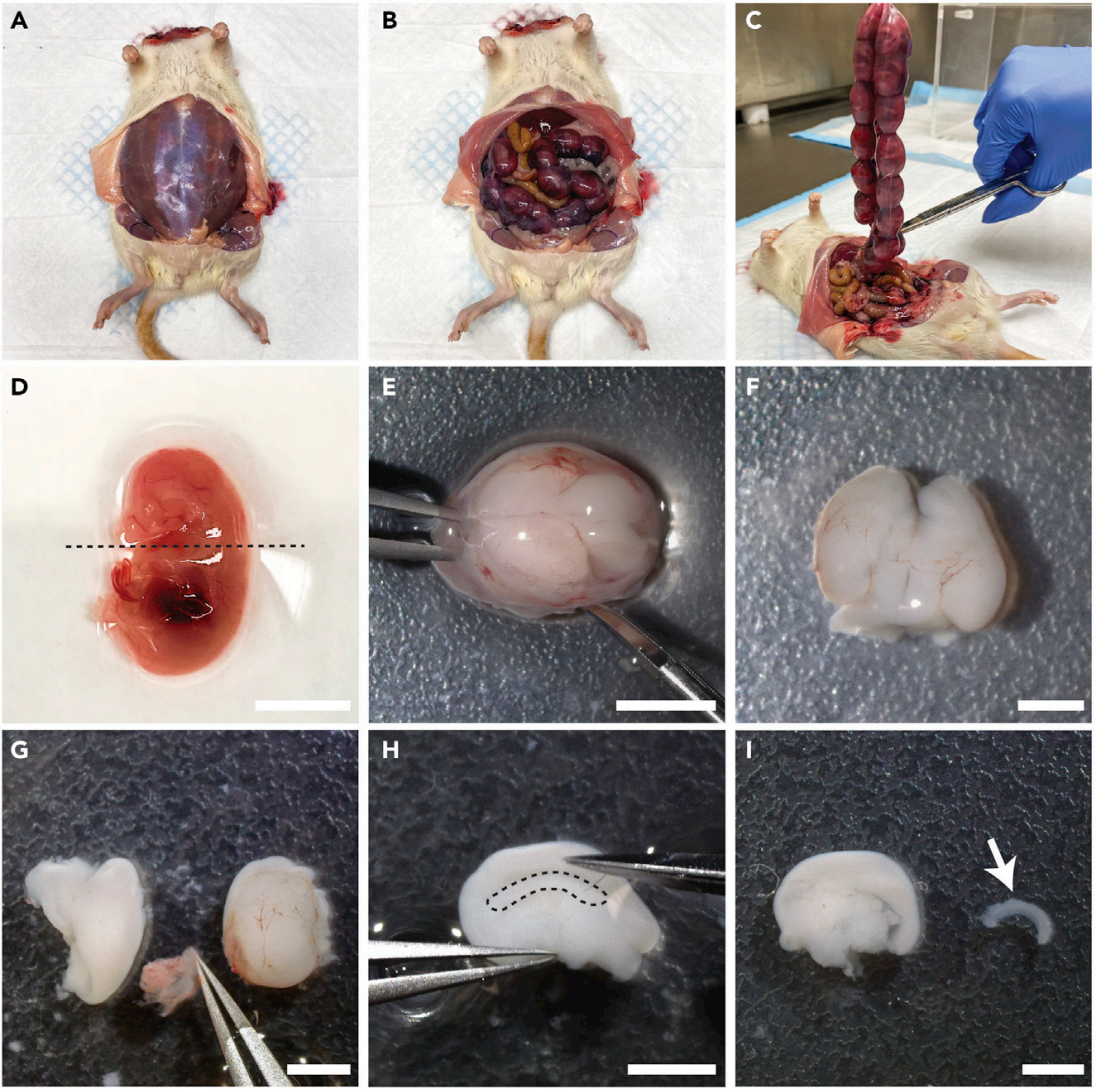
Preparation and hippocampal dissection of E18 rat embryos (A) Euthanized and decapitated E18 timed-pregnant rat with the abdominal fascia exposed. (B) Incision to open the abdominal cavity to expose the uterus. (C) Removing the uterus containing the prenatal pups. (D) Lateral cut to remove the head. Scale bar, 1 cm. (E) Incision to the mid-sagittal plane of the skull to expose the brain. Scale bar, 0.5 cm. (F) E18 brain with intact hemispheres; brain stem was removed. Scale bar, 0.25 cm. (G) Right and left hemispheres of the brain. The meninges are removed from the hemisphere on the left but not the right. Scale bar, 0.25 cm. (H) Interior of the brain with the hippocampus outlined in black. Scale bar, 0.25 cm. (I) Dissected hippocampus (see arrow). Scale bar, 0.25 cm.
4. Using a fresh pair of scissors and forceps, make an incision to open the abdomen and cut to expose the abdominal cavity (Figure 1B).
5. Using a fresh pair of scissors and forceps, remove the uterus and place it in a 15 cm dish containing ice-cold HBSS (Figure 1C).

***Note:*** Use fresh pairs of scissors and forceps to prevent contamination (troubleshooting 1).

1. Euthanize an E18 timed-pregnant rat using methods outlined in an institution-approved protocol, such as CO_2_ euthanasia or decapitation.
2. Spray the ventral area of the female rat with 70% ethanol and make a transverse incision between the dermal layer and abdominal fascia.
3. Using the scissors and forceps, separate the layers and cut to expose the abdominal fascia (Figure 1A).

6. Remove the embryos from the uterus and place them into a 10 cm dish that contains ice-cold HBSS.
7. Separate and place the heads into a new 10 cm dish that has ice-cold HBSS (Figure 1D). Keep the plate on ice.

***Note:*** Placing the specimen into new HBSS helps wash away residue and blood.

***Note:*** Make an even and straight cut when separating the head. This step is vital as the head must balance on the cut surface.

### Hippocampal dissection

**Timing: 60 min**

This section details the careful removal of hippocampi from dissected E18 rat embryo brains. The dissection should be performed efficiently under a stereo or dissecting microscope. Minimizing time spent dissecting the hippocampi will produce higher yields of viable cells.

8. Place cold HBSS into the lid of a 10 cm dish and add the embryo heads.
9. With the head in the prone position, make an incision into the mid-sagittal plane of the head from posterior to anterior using vannas scissors (Figure 1E).
10. Peel back the cranium. Using a closed pair of vannas scissors, gently scoop under the brain in an anterior to posterior direction to remove it from the skull. Cut the brain stem to separate the brain (Figure 1F).
11. Make a sagittal cut to separate the right and left hemispheres.
12. Remove the meninges from the brain’s surface using two fine-pointed forceps (Figure 1G). The meninges contain blood vessels, making them appear red in color. It is easiest to remove starting from the exterior, working towards the brain’s interior.
13. With the interior hemisphere facing up, remove the hippocampus using vannas scissors. The hippocampus is located below the cortex and appears dark and moon-shaped (Figures 1H and 1I).
14. Use a P1000 pipette to transfer hippocampi to the 15 mL conical containing ice-cold HBSS.

***Note:*** It is fastest to work in batches of 4–6 brains, completing the same step for each brain before proceeding to the next step (troubleshooting 2).

***Note:*** It is essential to use ice-cold HBSS. The tissue becomes tacky, fragile, and challenging to work with when warm.

### Hippocampal tissue dissociation and plating

**Timing: 6 h**

This section contains steps for the trypsinization and trituration of hippocampi. Once the tissue is dissociated, cells are plated on the glass surface of PLL-coated imaging dishes. These steps should be performed in a Biosafety cabinet.

15. Remove HBSS from the 15 mL conical with a serological pipet. Try not to disturb the hippocampi.
16. Add 4.5 mL of warm HBSS and 0.5 mL of 2.5% trypsin. Invert the conical once and incubate in a 37°C water bath for 10 min.
17. Stop digestion by removing the trypsin solution using a 10 mL serological pipet.
18. Add 10 mL of warmed AM, allow hippocampi to settle (approximately 30 s), and remove media using a 10 mL serological pipet.
19. Repeat step 18 two more times.
20. Add 1 mL of AM and triturate tissue with a fire-polished Pasteur pipette. Try not to introduce air bubbles. Proceed until the media is cloudy and tissue pieces are no longer visible (∼10–20 times).

***Note:*** Fire-polished Pasteur pipettes have smoother edges and a narrowed tip which help break up tissue more efficiently while reducing cell damage.

21. Dilute 10 μL of cell suspension with 10 μL of 0.4% Trypan Blue Stain. Determine the cell viability and count using an automated counter.

***Alternatives:*** The cell count can also be determined using a hemacytometer.

**CRITICAL:** Trypan blue is a carcinogen and may cause cancer. Wear appropriate PPE when working with the solution.

22. Plate 1.25 × 10^5^ cells onto the pre-coated/washed glass surface of 35 mm MatTek dishes containing warmed AM. Add dropwise, covering the entire glass surface.
23. Keep cultures at 5% CO_2_ and 37°C for ∼2–5 h to provide adequate time for cells to settle and attach to the glass surface.
24. Ensure cells have adhered through visual inspection using a light microscope. Upon confirmation, replace media with MM and maintain cultures at 37°C and 5% CO_2_.

### Maintaining hippocampal cultures

**Timing: 6 d**

Hippocampal cultures are maintained by performing media exchanges every few days. In addition, AraC is added to reduce non-neuronal cell growth the day after plating. These steps should be performed in the Biosafety cabinet.

25. The day after plating, or 1 day *in vitro* (DIV), add 1 μM AraC to cultures to prevent the proliferation of non-neuronal cells. It is best to dilute AraC in a small amount of MM (∼200 μL) and add it dropwise to the dish. Gently mix to ensure there is an even distribution of AraC.

***Note:*** Adding AraC the day after plating reduces the number of non-neuronal cells without affecting neuronal growth or survival. If performing studies on aged cultures, it is best to continue adding AraC during feedings.

26. Every 3–4 days, replace ¼ of culture media with equilibrated MM (i.e., media that has been warmed to 37°C and equilibrated to 5% CO_2_ for a minimum of 30 min).

***Note:*** Neurons are sensitive. Minimize stress by working quickly when handling cells outside the CO_2_ incubator (troubleshooting 2).

### Transient transfection of cultured neurons

**Timing: 2 h**

Lipofectamine 2000 is used to transiently transfect DNA plasmids. All transfection steps must be performed in the Biosafety cabinet.

27. At 6 DIV, check the quality of neuron cultures to ensure viability for transfection (Figure 2).

**Figure 2:**
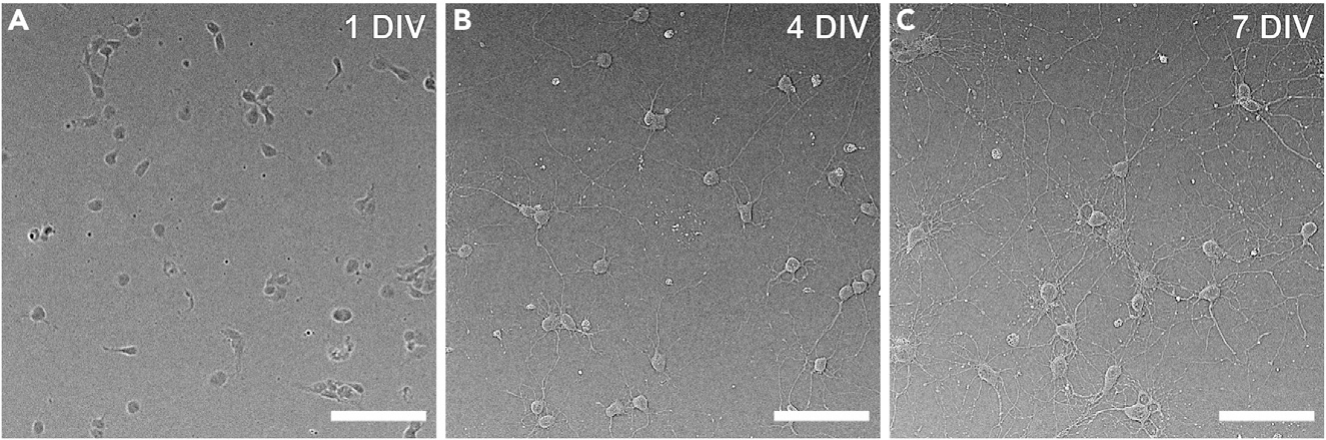
Micrograph of primary hippocampal neurons (A–C) Representative images of primary hippocampal neurons at one (A), four (B), and seven (C) DIV. Scale bar, 50 μm.
28. For each imaging dish, remove and save media for later use. Replace with fresh equilibrated MM.
29. For each transfection, prepare two separate mixtures in sterile microcentrifuge tubes and incubate at room temperature (20°C–22°C) for 5 min (Figures 3A and 3B).
  a. Tube 1: 150 μL Neurobasal media and DNA (0.35 μg Mito-SNAP, 0.4 μg Halo-OPTN, and 0.4 μg LAMP1-EGFP). Mito, sequence from COX8; OPTN, Optineurin; LAMP1, Lysosome Associated Membrane Protein 1.
  b. Tube 2: 150 μL Neurobasal media and 4 μL Lipofectamine 2000.

**Figure 3:**
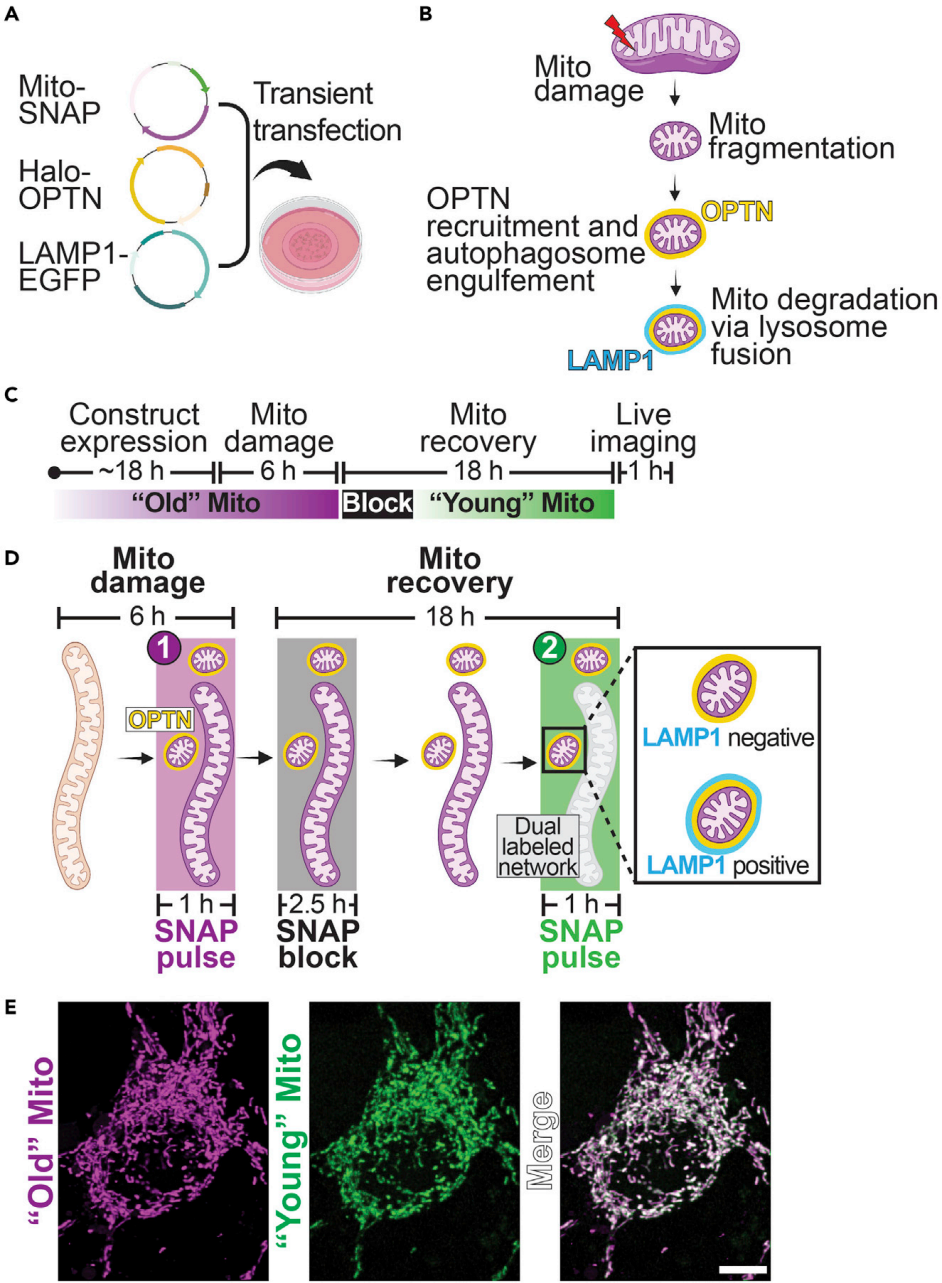
Mitochondrial damage and SNAP pulse-chase paradigm (A) Schematic of plasmids used to transiently transfect primary hippocampal cultures. (B) Schematic of mitochondrial damage. Antioxidant deprivation initiates mitochondrial damage and fragmentation. OPTN is recruited to damaged mitochondria and facilitates engulfment into an autophagosome, which fuses with a lysosome for downstream acidification to degrade the damaged organelle fully. (C and D) Timeline (C) and schematic (D) of the mitochondrial pulse-chase experiment. The day after transfection, mitochondria are damaged for 6 h and labeled with the first SNAP pulse (“Old” Mito, magenta). Next, a non-fluorescent SNAP Block was added for 2 h to saturate the remaining SNAP binding sites, and neurons were left for ∼16 h. Before imaging, a second SNAP pulse (“Young” Mito, green) was performed. (E) Representative images of “Old” and “Young” mitochondrial populations. The dynamic mitochondrial network is dual labeled (white). Scale bar, 5 μm.

***Note:*** According to the manufacturer, DNA-Lipofectamine complexes can be added to the culture medium in the presence or absence of serum/antibiotics. The manufacturer recommends using Opti-MEM Reduced Serum media for transient transfections. However, we found no difference in the transfection efficiency using Neurobasal media.

***Note:*** Use an endotoxin-free DNA Maxi Kit to prepare concentrated and high-quality DNA plasmids. If dissolving plasmids in TAE, be cautious when working with low DNA concentrations, as too much EDTA can decrease transfection efficiency.

30. Add Tube 2 to Tube 1, gently mix by pipetting, and incubate at 20°C–22°C for 20 min.

***Note:*** The manufacturer recommends adding Tube 1 to Tube 2 and incubating for 5 min. We found no difference in the transfection efficiency using our method. In addition, the increased incubation time allows for more DNA-Lipofectamine complexes to form.

31. Add transfection complexes dropwise to neuron cultures and incubate at 37°C in 5% CO_2_ for 45 min.
32. During the incubation, add equal parts of saved media from step 28 with fresh MM and equilibrate at 37°C in 5% CO_2_.
33. After 45 min, replace the neurons’ media with the conditioned media from step 32.

***Note:*** Transient transfection should be completed ∼18 h before pulse-chase labeling (troubleshooting 3).

### Mitochondrial damage and SNAP pulse-chase labeling assays

**Timing: 2 d**

Within this protocol, mitochondrial damage is induced via antioxidant deprivation using the AOF MM, which initiates mild oxidative stress and results in low levels of mitochondrial damage. Mitochondria are damaged for 6 h while SNAP and Halo labeling typically require 1 h (Figures 3C–3E; troubleshooting 4). Thus, labeling occurs during the final hour of the damaging incubation period. The ligand is added for 30 min, followed by two quick washes and a 30-min washout. The first SNAP pulse identifies “Old” mitochondria, while the second SNAP pulse labels “Young” mitochondria (Figures 3C and 3D). For SNAP-Cell Block, the ligand is added for 120 min, followed by two quick washes and a 30-min washout. To complete all required washes, it is necessary to warm ample media to 37°C in the 5% CO_2_ incubator. Mitochondrial damaging and pulse-chase labeling assays must be performed in the Biosafety cabinet.

34. 18–24 h after transfection, remove media from each imaging dish and reserve for later. Save media separately in sterile microcentrifuge tubes.
35. Mitochondrial-damaging assay.
  a. For each experimental plate, induce mitochondrial damage by adding 2 mL of AOF MM. For the control condition, add 2 mL of regular MM.
  b. Place in the 5% CO_2_ incubator at 37°C for 6 h.
36. 1^st^ SNAP pulse.
  a. During the final hour of the mitochondrial-damaging assay, add 100 nM of SNAP JF549cp and 100 nM of Halo JF646x ligands to each plate. Thoroughly mix and incubate for 30 min in the 5% CO_2_ incubator at 37°C (troubleshooting 5).
  b. Wash plates twice with 2 mL of media. Add 2 mL of media and place dishes back in the 5% CO_2_ incubator at 37°C for a final 30-min washout.
    i. Wash with AOF MM for treated plates and regular MM for control plates.
37. End mitochondrial-damaging assay and 1^st^ SNAP pulse.
  a. After 6 h treatment, aspirate AOF MM and add 2 mL of regular MM. Perform the same steps with the control plate for consistency, removing media and adding 2 mL of MM.
38. SNAP Block.
  a. Add 1 μM of SNAP-Cell Block to each plate and thoroughly mix.
  b. Incubate for 120 min in the 5% CO_2_ incubator at 37°C.
  c. Wash plates twice with 2 mL of MM. Add 2 mL of MM and place the dishes back in the 5% CO_2_ incubator at 37°C for a final 30-min washout.

***Note:*** SNAP-Cell Block is a membrane-permeable SNAP ligand that does not fluoresce. This step is necessary to saturate the remaining SNAP binding sites following the 1^st^ SNAP pulse.

39. Add conditioned media from step 34 back to imaging dishes and place in the 5% CO_2_ incubator at 37°C for ∼16 h.
40. 2^nd^ SNAP Pulse.
  a. 23 h after beginning the mitochondrial-damaging assay, add 1 μM of SNAP-Cell 430 ligand.
    ***Note:*** SNAP-Cell 430 is a costly reagent. To be consistent with the 2 mL volume used in the first SNAP pulse, 4 μL of SNAP-Cell 430 are required. Alternatively, dilute 1 μL SNAP-Cell 430 in 500 μL of MM. Be sure the neurons are entirely covered with media, as low liquid volumes could affect the relative oxygen tension (troubleshooting 4).
  b. Incubate for 30 min in the 5% CO_2_ incubator at 37°C.
  c. Wash plates twice with 2 mL of MM. Add 2 mL of MM and place the dishes back in the 5% CO_2_ incubator at 37°C for a final 30-min washout.
41. Aspirate media and add 2 mL of MM. Cells are now ready for imaging; this should be 24 h after initiating mitochondrial damage.

***Alternatives:*** A selection of SNAP and Halo fluorescent labels are available. The ligands used here could be substituted based on experimental conditions and microscope laser line setup.

### Live-cell confocal imaging

**Timing: 2–3 h**

Before imaging, turn on the temperature and CO_2_ controller and allow conditions to stabilize (∼1 h). If using a microscope with a 5% CO_2_ environmental chamber, image neurons in MM; if not, use a media that can maintain cells in ambient CO_2_, such as Hibernate E Low Fluorescence by BrainBits supplemented with B27. When performing quantitative live-cell imaging, design appropriate configurations where all channels have good signal-to-noise and avoid pixel saturation, fluorophore crosstalk, and accidental FRET.

**Figure 4:**
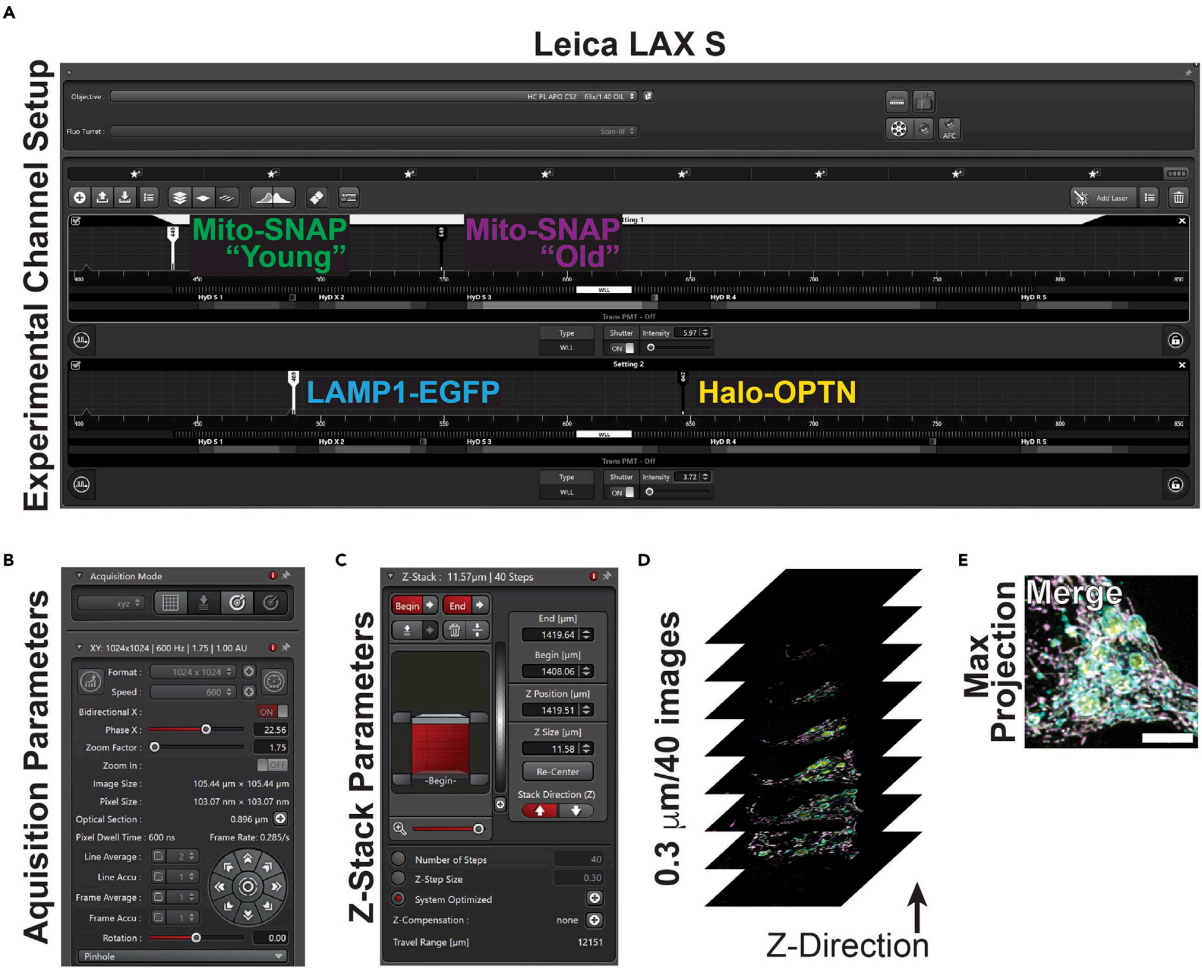
Acquiring Z-stack images of hippocampal neurons (A) Leica LAX S software with the experimental channel setup to image SNAP 430, EGFP, SNAP JF546cp, and Halo JF646x. (B) Acquisition parameters. (C) Z-stack parameters. (D) Diagram of a Z-stack of the soma of the hippocampal neuron. (E) Max projection of the Z-stack from panel D. Scale bar, 5 μm.

42. Laser-scanning confocal setup using a Leica STELLARIS 8 equipped with a 63×/1.40 oil objective and LAS X Software (Figure 4A).
  a. Setting 1: SNAP-Cell 430, Excitation 440 nm, Emission 450–486; SNAP JF549cp, Excitation 549, Emission 559–637.
  b. Setting 2: EGFP, Excitation 489, Emission 499–543; Halo JF646x, Excitation 647, Emission 657–750.
  c. Acquire Z-stack images in “Sequential Scanning” mode.

**CRITICAL:** Use “Sequential Scanning” mode and built-in crosstalk function in LAS X to determine fluorophore crosstalk (troubleshooting 6). The spectra of the fluorophores used in this protocol are close to each other. Thus, the detection window of SNAP-Cell 430 was narrowed to avoid crosstalk with EGFP.

43. Acquire images at 1024 × 1024 format (0.103 μm/pixel; image size 105.10 μm × 105.10 μm), 0.3 μm step size (averaging 35–50 steps per image), using bidirectional scanning, 600 Hz scan speed, zoom factor of 1.75, and line average of 2. The pinhole size was 1 AU (Figures 4B–4E and Methods video S1).
  a. Use the Over/Under look-up table to avoid pixel saturation throughout all Z-planes and for all channels (troubleshooting 6).
44. Deconvolve images using LIGHTENING Process with adaptive strategy, a refractive index of 1.33, and water as the mounting medium. All channels used 20 iterations, an optimization of 1, a regularization parameter of 0.05, and “Very High” smoothing. Contrast enhancement and cut-off were set to “Auto.”
45. Save images files for subsequent analysis.

***Note:*** Pixel misalignment may occur when imaging and can be corrected during post-processing. Perform the correction before the analysis, as it can affect marker colocalization (troubleshooting 7).

***Alternatives:*** Imaging times are reduced using media that maintains cells in ambient CO_2_. To determine how long a plate can be kept in ambient CO_2_, consult the manufacturer for the buffering capacity.

Methods video S1. Confocal Z-stack to monitor mitochondrial turnover, related to steps 42 and 43 Representative neuron following mitochondrial pulse-chase labeling, illustrating distinct “aged” mitochondrial populations. Live-cell confocal imaging was used to take a Z-stack through the soma of a primary hippocampal neuron. Scale bar, 5 μm.

### Quantitative data analysis

**Timing: 2 h**

We use spectrally distinct fluorophores to identify “Old” and “Young” mitochondria. To distinguish between these two populations, it is essential to note which channel corresponds to each fluorophore when analyzing the data. The “Rectangle” and “Line” functions in Fiji are used to identify OPTN-positive “Old” mitochondria and quantify the fluorescence intensity.

46. Open image files in Fiji.
  a. Channel 1: “Young” Mito-SNAP, SNAP-Cell 430.
  b. Channel 2: “Old” Mito-SNAP, SNAP JF549cp.
  c. Channel 3: LAMP1-EGFP.
  d. Channel 4: Halo-OPTN, Halo JF646x.
47. Identify OPTN-positive “Old” mitochondria (Figure 5A–5C).
  a. Use the “Channels Tool” to visualize “Young” Mito-SNAP (SNAP-Cell 430), “Old” Mito-SNAP (SNAP JF549cp), and Halo-OPTN (Halo JF646x) channels.

**Figure 5:**
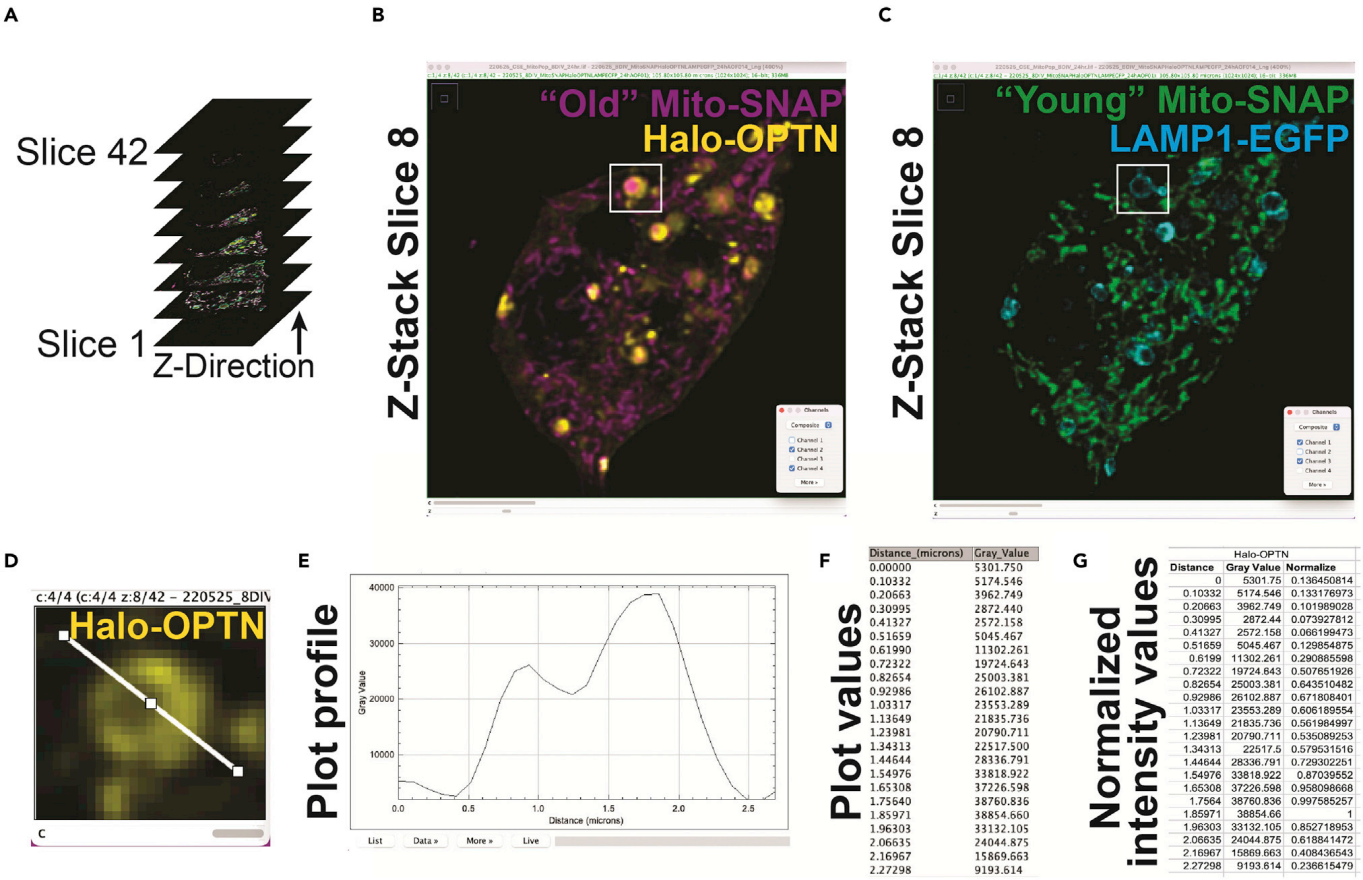
Identification of “Old” sequestered mitochondria (A) Diagram of a Z-stack of a hippocampal neuron (from Figure 4D). (B and C) Slice 8 of a hippocampal neuron illustrating “Old” Mito-SNAP and Halo-OPTN (B) or “Young” Mito-SNAP and LAMP1-EGFP (C). Each Z-plane is visualized to identify OPTN-positive “Old” mitochondria manually. A white box highlights OPTN-positive “Old” mitochondrion that is negative for “Young” Mito-SNAP but positive for LAMP1-EGFP. (D) Magnification of panel B illustrates Halo-OPTN. A line is drawn using the “Straight Line” tool to generate a line scan. (E–G) Graphing the plot profile of the line from panel D (E) to obtain the plot fluorescence intensity values (F). Values are normalized (G) and graphed for each channel (see Figure 6).
  b. Starting with the first Z-step, manually move through the Z-stack to identify Halo-OPTN rings. In a single Z-plane, these events are visualized as OPTN rings encircling fragmented mitochondria.
  c. For each OPTN ring event, determine whether they are dual labeled with “Young” and “Old” Mito-SNAP or only labeled with “Old” Mito-SNAP.
    i. “Young” and “Old” mitochondria-positive events: colocalization of SNAP-Cell 430 and SNAP JF549cp mitochondrial markers within the OPTN ring.
    ii. “Old” mitochondria-positive events: only SNAP JF549cp mitochondrial marker within the OPTN ring, where no SNAP-Cell 430 fluorescence signal is present within the OPTN ring.
      ***Note:*** It is rare to observe OPTN ring events only labeled with “Young” Mito-SNAP, and they should be excluded from this analysis.
  d. Once a clear, well-defined event has been identified, use the “Rectangle” function to draw a box. Create an overlay specific to the Z-stack slice. Only mark events that are OPTN-positive “Old” Mito-SNAP for further analysis; omit OPTN events that are dual labeled with “Young” and “Old” Mito-SNAP.
    ***Note:*** Use the “Channels Tool” and “Brightness & Contrast Tool” to help adjust the brightness, toggle between channels to identify events, and view multiple Z-planes around the OPTN ring for confirmation.
  e. Save each image file after visualizing all events in the cell.
48. Determine whether OPTN events containing “Old” mitochondria are positive or negative for LAMP1.
  a. Use the “Channels Tool” to visualize “Old” Mito-SNAP (SNAP JF549cp), Halo-OPTN (Halo JF646x), and LAMP1-EGFP channels.
  b. Use the boxes created in step 47 to locate OPTN ring events.
  c. Score OPTN-positive “Old” mitochondria for the presence or absence of LAMP1-EGFP.
    i. LAMP1-positive events: colocalization of LAMP1-EGFP with Halo-OPTN, visualized as overlapping rings in a single Z-plane. Only clear, well-defined LAMP1 rings are included in the analysis.
    ii. LAMP1-negative events: no LAMP1-EGFP fluorescence signal or there is no colocalization of LAMP1-EGFP with Halo-OPTN around damaged mitochondria. LAMP1-EGFP puncta are scored as LAMP1-negative.
49. Quantify the ratio of “Old” mitochondria positive and negative for LAMP1.
  a. Divide the number of LAMP1-positive events by the total number of OPTN-positive “Old” mitochondria.
  b. Divide the number of LAMP1-negative events by the total number of OPTN-positive “Old” mitochondria.
50. Generate representative line scans for OPTN-positive “Old” mitochondria events.
  a. Use the “Line” function to draw a line (width = 1) through the Halo-OPTN ring (Figure 5D). The line should be drawn through a representative area, avoiding high-fluorescence artifacts and saturated pixels (troubleshooting 6).
  b. For each channel, use “Plot Profile” and “List” to obtain the fluorescence intensity values for the drawn line (Figures 5E and 5F).
  c. Use the “Rectangle” function to draw a box and randomly measure the fluorescence intensity of the image background using “Measure” in the “Analyze” tab. Repeat in multiple representative areas and calculate the average background intensity for each channel.
  d. Correct the fluorescence signal for each channel by subtracting the average background intensity from the measured fluorescence intensity values from the line scan.
  e. For each channel, normalize the intensity values by dividing each value by the overall highest intensity value for that channel. Normalization sets the highest value to 1 and additional values to less than one. (Figures 5G,6A, and 6B).
51. Data can be presented as bar graphs, scatter plots, or pie charts with representative images.

**Figure 6:**
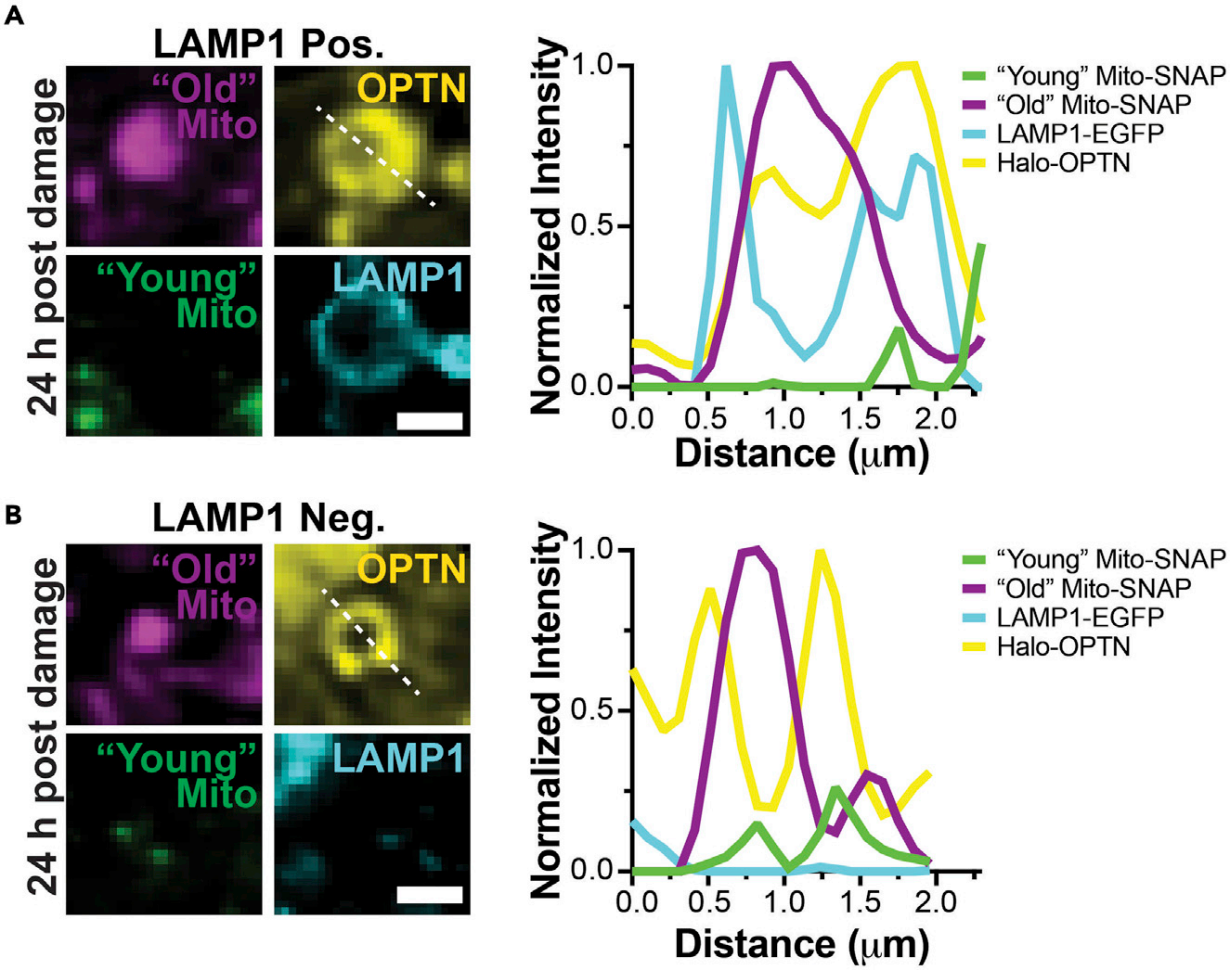
A population of aged mitochondria has yet to be degraded 24 h post mitochondrial damage (A and B) Representative images of neurons 24 h post damage illustrating OPTN sequestered “Old” mitochondria that are positive (A) or negative (B) for LAMP1 (lysosome marker). These mitochondria are negative for “Young” Mito-SNAP, illustrating that these organelles were sequestered for hours. Line scans are shown to the right. Scale bar, 5 μm.

## Expected outcomes

Cellular antioxidants, such as superoxide dismutase, catalase, and glutathione, protect against oxidative damage by maintaining the oxidative balance. In this protocol, we described a mitochondrial damaging paradigm where antioxidant deprivation (AOF) induced mild oxidative stress (Joselin et al., 2012; Evans and Holzbaur, 2020a). Removing antioxidants from the cell culture media resulted in low levels of reactive oxygen species sufficient to damage mitochondria and initiated the mitophagy pathway. Within this pathway, autophagy receptors bind to phospho-ubiquitinated mitochondrial fragments and recruit autophagosomes to sequester and degrade damaged organelles via lysosome fusion (Evans and Holzbaur, 2020b; Youle and Narendra, 2011). Mitophagy events were identified by the translocation of OPTN, a known autophagy receptor, while lysosomal fusion was monitored using LAMP1 (Figure 3B).

Here, we exploited a mitochondrially targeted SNAP-tag construct and SNAP ligands to monitor long-term mitochondrial degradation in primary hippocampal neurons (Grimm et al., 2015, 2017). Pulse-chase labeling allowed for the visualization of distinct “Old” and “Young” mitochondrial populations (Figure 3). The “Old” mitochondria were labeled during the first SNAP pulse and comprised Mito-SNAP expressed from the time of transfection until SNAP Block labeling (∼24 h). Meanwhile, the “Young” mitochondria were labeled during the second SNAP pulse and represented Mito-SNAP expressed from the time after SNAP Block until imaging (∼22 h; Figure 3C). Since mitochondrial damage via AOF treatment coincided with the first SNAP pulse, we identified mitophagy events where OPTN translocated to “Old” fragmented mitochondria. Visualizing OPTN-positive “Old” mitochondria that were negative for the “Young” mitochondria marker demonstrates that these “aged” mitochondria were sequestered and engulfed before the addition of the second SNAP pulse.

Next, we identified OPTN-positive “Old” mitochondria that were negative for “Young” mitochondria and quantified the ratio of events that were LAMP1-negative or LAMP1-positive. In control and AOF conditions, the majority of “Old” mitochondria were LAMP1-positive. Colocalization of “Old” mitochondria with LAMP1 illustrates that the autophagosome containing the damaged mitochondria fused with the lysosome for degradation. Surprisingly in both treatment groups, a population of OPTN-positive “Old” mitochondria was LAMP1-negative 24 h after initial damage, suggesting that this population of “Old” damaged mitochondria failed to fuse with lysosomes to undergo degradation (Figure 6). Using this pulse-chase paradigm, we demonstrated that dysfunctional mitochondria persist for hours to days following mitochondrial insult. Thus, the degradation of engulfed mitochondria takes much longer than the minutes-to-hours time scale that was previously reported (Ashrafi et al., 2014; Hsieh et al., 2016). Inefficient degradation of damaged organelles may contribute to decreased neuron viability and neurodegeneration.

Our protocol could investigate mitochondrial turnover in disease backgrounds using primary cultures from OPTN transgenic knockout or mutant mice. Mutations in OPTN are linked to amyotrophic lateral sclerosis (ALS), a neurodegenerative disease caused by the gradual degeneration of motor neurons (Maruyama et al., 2010; Maruyama and Kawakami, 2013). In cell-based assays, expression of OPTN-linked mutations affected mitochondrial network health and either decreased the efficiency or disrupted the mitophagy pathway (Wong and Holzbaur, 2014; Evans and Holzbaur, 2020a). Therefore, our protocol could be easily adapted to study mitochondrial turnover in disease-related contexts. Additionally, modifying the timeframe between the two pulses would enable monitoring of the mitochondria over extended periods.

While this protocol was designed for primary hippocampal neurons, we anticipate that it will be easily adapted for other primary culture systems and non-neuronal cell lines. SNAP-tag cloning vectors have been used to generate plasmids encoding fusion proteins localized to the nucleus, endoplasmic reticulum, and many other cellular structures. Thus, this SNAP pulse-chase labeling protocol could be used to monitor the long-term turnover of additional organelles.

## Limitations

Primary hippocampal neuron cultures are isolated and do not have the same accompanying cells or architecture as *in vivo*, which could impact physiology. However, neuron cultures are routinely used to investigate numerous aspects of the cell biology of the neuron. In this protocol, live confocal microscopy was used to study mitochondrial damage. Care must be taken with the laser power and exposure time when imaging to prevent phototoxicity. Transient transfection can produce a range of protein concentrations that vary from cell to cell. Thus, it is vital to avoid high-expressing cells as it may alter the biology. Furthermore, the time scale of mitochondrial turnover observed is specific to primary hippocampal neurons. We expect the rates of mitochondrial turnover observed here to be consistent across neuronal cell types, but this has yet to be directly demonstrated.

## Troubleshooting

### Problem 1

Sample contamination during embryonic dissection and hippocampal isolation. Step 5.

### Potential solution

There are multiple places where contamination can occur. It is best to wipe down all dissection surfaces and tools with 70% ethanol before starting the dissection. Animal-generated contaminants can be a significant source of contamination. Use a clean pair of scissors and forceps for each step of the embryo isolation during the dissection. Switching to a fresh pair of gloves between working with animals and isolating hippocampi can help prevent animal-generated contamination.

### Problem 2

Poor survival or quality of primary hippocampal cultures. Steps 8 and 26.

### Potential solution

Timed-pregnant female rats typically have 8–12 embryos. It is essential to inspect embryos when dissecting to ensure health. Avoid using too small/large or discolored embryos, as they can result in neuron death when plated. Performing successful dissections and obtaining high-quality cultures is skill-dependent (Figure 1). The survival and quality of cultures also increase with practice and repetition.

Additional factors that can affect the quality or survival of primary hippocampal cultures include 1) dissecting in a timely manner to maintain tissue quality, 2) performing procedures (e.g., transfections, feedings, labeling) efficiently to decrease the amount of time primary cultures spend outside the incubator, 3) using fresh or recently made media, and 4) carefully monitoring the lot-to-lot variability of media components (e.g., GlutaMAX, B27, and Neurobasal media).

### Problem 3

Low transfection efficiency of primary hippocampal neurons. Step 27.

### Potential solution

If experiencing low transfection efficiency, increase the DNA concentration or the amount of Lipofectamine 2000 used. In this protocol, 1.1 μg total DNA and 4 μL Lipofectamine 2000 were used for transient transfection, which is in the lower range for concentrations specified by the manufacturer. Additionally, alternative transfection reagents or techniques may be used.

Additional constructs could be used to study mitochondrial turnover. If generating new SNAP-tagged constructs, it is important that the tagged protein 1) is routinely used, 2) is specifically targeted to mitochondria, and 3) does not affect non-target pathways. While highly unlikely, low expression of Mito-SNAP could affect the feasibility of the protocol. Alternatively, Mito-Halo or Mito-Timer constructs could be used in place of Mito-SNAP.

### Problem 4

Poor neuron survival following SNAP pulse-chase. Step 36.

### Potential solution

It is best to use prewarmed media to 37°C and equilibrated to 5% CO_2_ in an incubator. Gently add and remove media to the imaging dish’s side to avoid disturbing the neurons. We have found that adding the reserved media (step 34) back to the imaging dish after the SNAP block (step 39) helps with the viability of the neurons.

Moreover, the SNAP-Cell 430 labeling occurs in 500 μL of media to save on costly reagents. Low volumes of liquid could affect the relative oxygen tension. If researchers observe that the low volumes affect neuronal health, we recommend performing the SNAP labeling in 2 mL of media instead of 500 μL.

### Problem 5

Difficulty visualizing SNAP or Halo ligands. Step 36.

### Potential solution

Difficulty visualizing ligands is typically due to inadequate labeling. To ensure equal distribution, we have found it is best to premix ligands in a small volume of media and then add it to the imaging dishes. Higher concentrations of ligands can be used if necessary. In addition, store ligands in the dark and avoid using ligands past the recommended storage time.

### Problem 6

Fluorescence crosstalk, FRET, and image saturation. Steps 42 and 43.

### Potential solution

Select fluorophores with non-overlapping excitation and emission spectra to avoid fluorescence crosstalk and accidental FRET. We recommend using the “Sequential Scanning” Mode to separately excite individual fluorophores or pairs of fluorophores with non-overlapping spectra. Minimize the detection window to reduce the signal from other fluorophores or increase the number of settings to mitigate crosstalk. To check for the presence of fluorescence or accidental FRET, create new image parameters excluding one fluorophore while leaving the remaining laser lines intact. Comparing this null image with the image containing all laser lines will identify potential crosstalk generated while imaging. Several microscope software programs have built-in functions that determine the amount of crosstalk, such as Leica’s LAS X.

When acquiring images, it is critical to avoid pixel saturation. Saturated pixels can significantly alter the normalized fluorescence intensity for line scans. Use the Over/Under look-up table to identify pixel saturation, visualized as blue pixels. It is necessary to adjust the settings for each channel and scroll through the Z-planes to ensure there are no saturated pixels.

### Problem 7

Correcting image misalignment. Step 43.

### Potential solution

It is possible that pixel misalignment can occur and is most easily visualized when comparing SNAP-Cell 430 and SNAP JF549cp channels. The offset of the lasers and mirrors in the microscope’s optical path leads to image misalignment. To correct this, we recommend using optical beads to determine the relative alignment of the channels in X, Y, and Z. If the alignment is significantly off, we suggest having the microscope realigned. Minor misalignments can be corrected during post-processing. LAS X software can be used to fix misalignment in XY, but not Z. To alter the registration in X, Y, and Z, we recommend using the “MultiStackReg” plugin in Fiji.

## Resource availability

### Lead contact

Further information and requests for resources and reagents should be directed to and will be fulfilled by the lead contact, Chantell Evans (chantell.evans@duke.edu).

### Materials availability

This study did not generate new unique reagents. The recombinant DNA used in this study is available from the lead contact upon request.

## Data Availability

https://doi.org/10.7554/eLife.50260 Any additional information is available from the lead contact upon request.
